# Molecular mechanisms of simvastatin response in *Mesocestoides corti*: a proteomic approach to drug repurposing for larval cestodiases

**DOI:** 10.1186/s13071-026-07568-x

**Published:** 2026-07-09

**Authors:** Marina Monteiro Guedes, Jeferson Camargo de Lima, Jéssica Andrade Paes, Leo Kei Iwai, Karina Mariante Monteiro, Arnaldo Zaha, Henrique Bunselmeyer Ferreira

**Affiliations:** 1 Programa de Pós-Graduação em Biologia Celular e Molecular, Centro de Biotecnologia (CBiot), Universidade Federal do Rio Grande do Sul (UFRGS), Porto Alegre, RS, Brazil; 2 Laboratório de Genômica Estrutural e Funcional, CBiot, UFRGS, Porto Alegre, RS Brazil; 3 Laboratório de Biologia Molecular de Cestódeos, CBiot, UFRGS, Porto Alegre, RS Brazil; 4 Laboratório de Bioinformática e Bioquímica de Proteínas, Universidade do Vale do Taquari (Univates), Lajeado, RS Brazil; 5 Laboratório de Toxinologia Aplicada (LETA) e Centro de Toxinas, Resposta-Imune e Sinalização Celular (CeTICS), Instituto Butantan, São Paulo, SP Brazil

**Keywords:** Mevalonate pathway, Drug repurposing, Simvastatin, *Mesocestoides corti*

## Abstract

Larval cestodiases remain difficult to treat, highlighting the need for novel therapeutic targets. The mevalonate pathway, essential for isoprenoid biosynthesis and cellular homeostasis, represents a promising target in cestodes. Previous studies demonstrated that simvastatin impairs the viability and motility of *Mesocestoides corti* tetrathyridia *in vitro*. Here, we investigated the proteomic response of *M. corti* larvae to simvastatin exposure (60.29 and 100 µM) using DIA-based proteomics. A total of 3,378 proteins were identified, with 19 showing differential abundance across treatments. Proteomic and Gene Ontology enrichment analyses revealed dose-dependent alterations associated mainly with structural organization, metabolism, intracellular transport, and stress-related processes. Although no clear proteomic signature of cell death was detected, simvastatin induced adaptive responses consistent with compensatory remodeling in response to pharmacological stress. These findings support the biological relevance of the mevalonate pathway in cestode survival and reinforce HMG-CoA reductase as a potential target for drug repurposing strategies against larval cestodiases.

**Graphical Abstract:**

**Figure:**
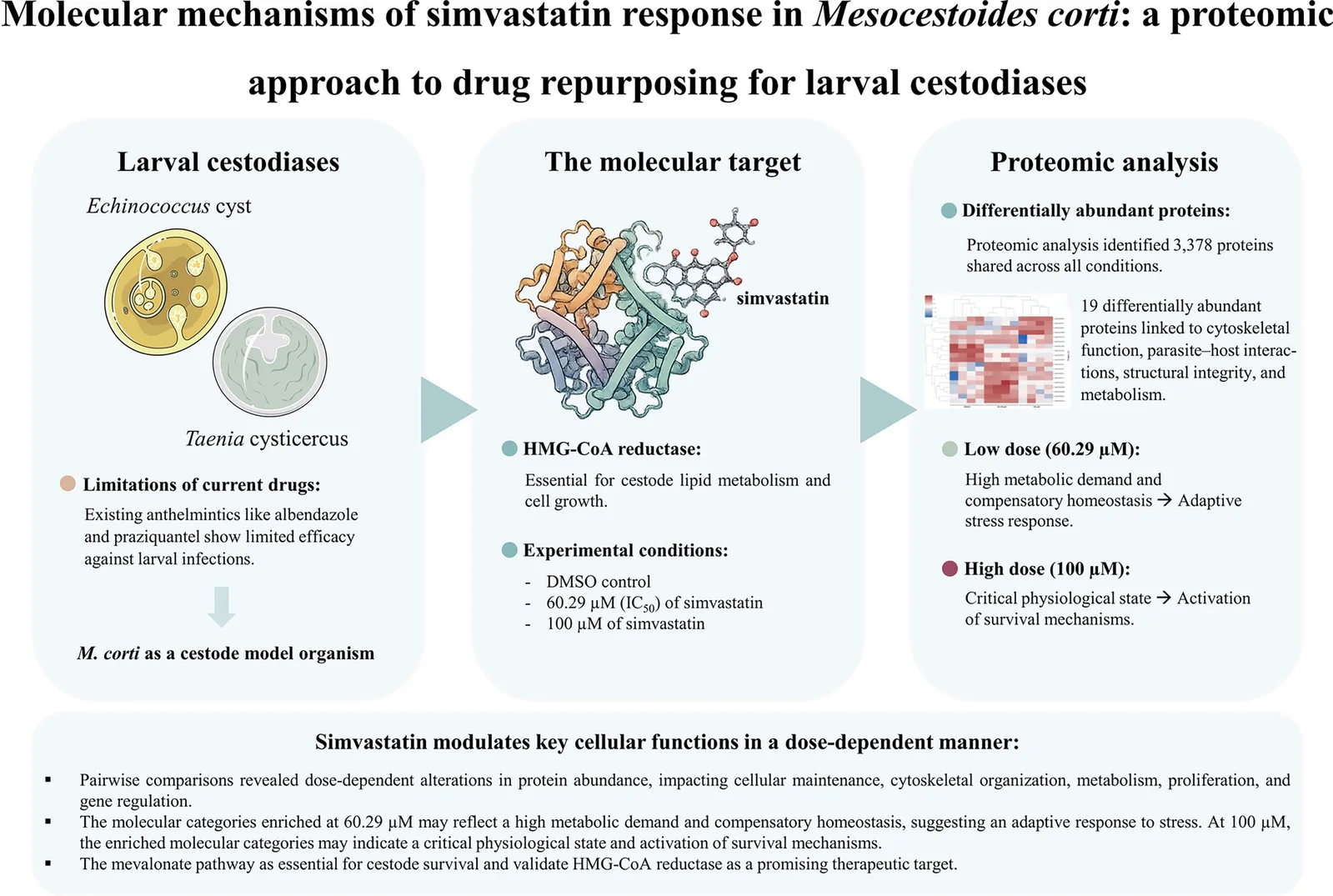

**Supplementary Information:**

The online version contains supplementary material available at https://doi.org/10.1186/s13071-026-07568-x.

Cestode larvae (metacestodes) are the etiological agents of severe zoonotic diseases, including cysticercosis, cystic echinococcosis (CE), and alveolar echinococcosis (AE) [1]. These infections are classified as neglected tropical diseases due to their global burden and limited therapeutic options [2]. Current treatment relies mainly on benzimidazoles and praziquantel, which show suboptimal efficacy against established infections [3–5], highlighting the need for new drug targets. Drug repurposing represents a promising strategy to accelerate therapeutic discovery [6]. The mevalonate pathway, in which HMG-CoA reductase plays a central role, is essential for isoprenoid biosynthesis and lipid-associated processes in helminths, despite their inability to synthesize cholesterol de novo [7–9]. Statins, a class of drugs that selectively inhibit HMG-CoA reductase, are clinically used to treat hyperlipidemia and are also under investigation for other therapeutic applications [10, 11]. We have previously shown that statins, particularly simvastatin, exert a potent *in vitro* effect against the larval stage (tetrathyridia, TTs) of the model cestode *Mesocestoides corti*, significantly reducing parasite viability and motility [12]. However, the molecular basis underlying these effects remains largely unknown. Therefore, the aim of the present study was to perform a comparative proteomic analysis of simvastatin-treated (60.29 and 100 µM) and untreated *M. corti* TTs to identify differentially expressed proteins and gain insight into the potential molecular pathways and biological processes associated with its antiparasitic activity.

*Mesocestoides corti* larvae (200 μL of TTs in 3 mL of medium) were cultured in RPMI 1640 medium at 37 °C under a 5% CO₂ atmosphere and exposed to simvastatin (60.29 µM, at the previously determined *in vitro* IC₅₀ [12] or 100 µM) for 72 h, using DMSO as a vehicle control. Four biological replicates per condition were used. After treatment, total protein extracts were prepared as previously described [13]. Mass spectrometry analyses were performed using an Orbitrap Exploris 480 instrument. Raw data were processed via DIA-NN [14] against the *M. corti* proteome (WormBase ParaSite [15]). Proteins identified with at least two unique peptides in at least two replicates were retained. Protein intensity values (MaxLFQ) were logarithmically transformed and normalized by Z-score. Consistency between biological replicates was confirmed by strong Pearson correlation coefficients (> 0.85). Box plots of the normalized distributions were used to assess data homogeneity. To assess the overall variability of the sample, Principal Component Analysis (PCA) was performed on the normalized dataset and imputed by the median. Proteins with differential abundance were assessed using ANOVA followed by unpaired t-tests adjusted for FDR (FDR < 0.05; |log₂FC|> 1). Proteomic analyses were conducted in Python (v3.11). Functional enrichment was performed using g:Profiler [16].

Data normalization and principal component analysis (PCA) indicated overall consistency among replicates and a moderate dose-dependent proteomic response to simvastatin, with greater divergence observed at 100 µM (Supplementary Fig. 1). Most identified proteins (3,378) were shared across all conditions, whereas only 19 displayed differential abundance between groups (Fig. 1A, B; Supplementary Tables 1 and 2). Differentially abundant proteins were broadly associated with structural organization, metabolism, intracellular transport, and stress-related processes. Condition-specific proteins were also detected, although these findings should be interpreted cautiously, as they may partly reflect stochastic variation or DIA detection sensitivity.

**Fig. 1:**
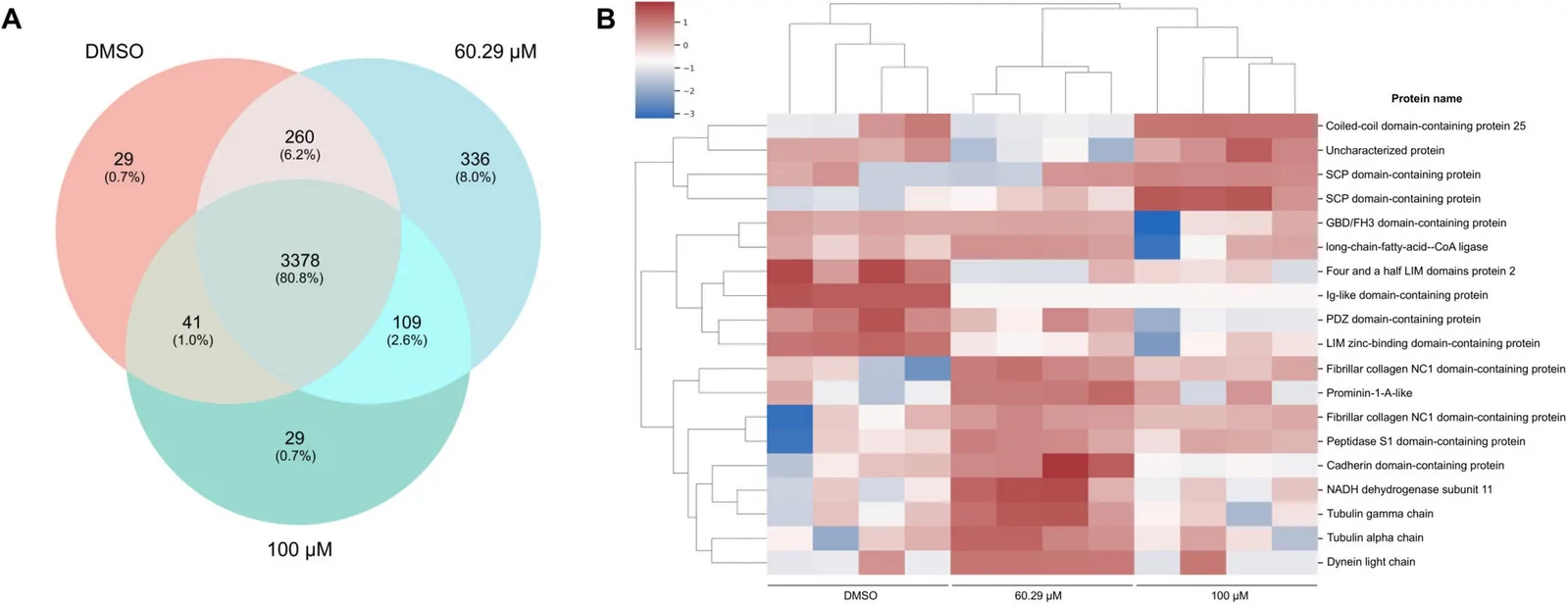
Proteomic identification and differentially abundant proteins upon simvastatin treatment in *M. corti*. **A** Venn diagram of proteins identified across experimental conditions: DMSO vehicle control, 60.29 µM simvastatin (IC₅₀), and 100 µM simvastatin. The numbers and percentages indicate the count of proteins identified exclusively in one condition or shared between conditions. The core proteome of 3,378 proteins (80.8%) was common to all groups. **B** Heatmap depicting the abundance profiles (log₂-transformed and Z-score normalized intensities) of the 19 differentially abundant proteins (rows) across the three conditions (columns). Hierarchical clustering groups samples by condition and proteins by abundance similarity. The color scale represents relative protein abundance from low (blue) to high (yellow). Protein identifiers are from the *M.* *corti* WormBase ParaSite proteome

Differential abundance analysis identified a limited set of proteins modulated by simvastatin treatment (Fig. 2A; Supplementary Tables 3 and 4). These proteins were mainly associated with structural organization, intracellular transport, metabolic adaptation, and stress-related processes. Proteomic alterations were more pronounced at 100 µM, suggesting a dose-dependent response to mevalonate pathway inhibition. No evident enrichment of proteins directly associated with cell death pathways was observed. GO enrichment analysis identified dose-dependent functional alterations following simvastatin treatment (Fig. 2B; Supplementary Table 4). While the control group was predominantly associated with basal metabolic and protein-processing functions, treated groups showed enrichment of categories related to structural remodeling, metabolism, intracellular transport, and adaptive stress responses, particularly at 100 µM.

**Fig. 2:**
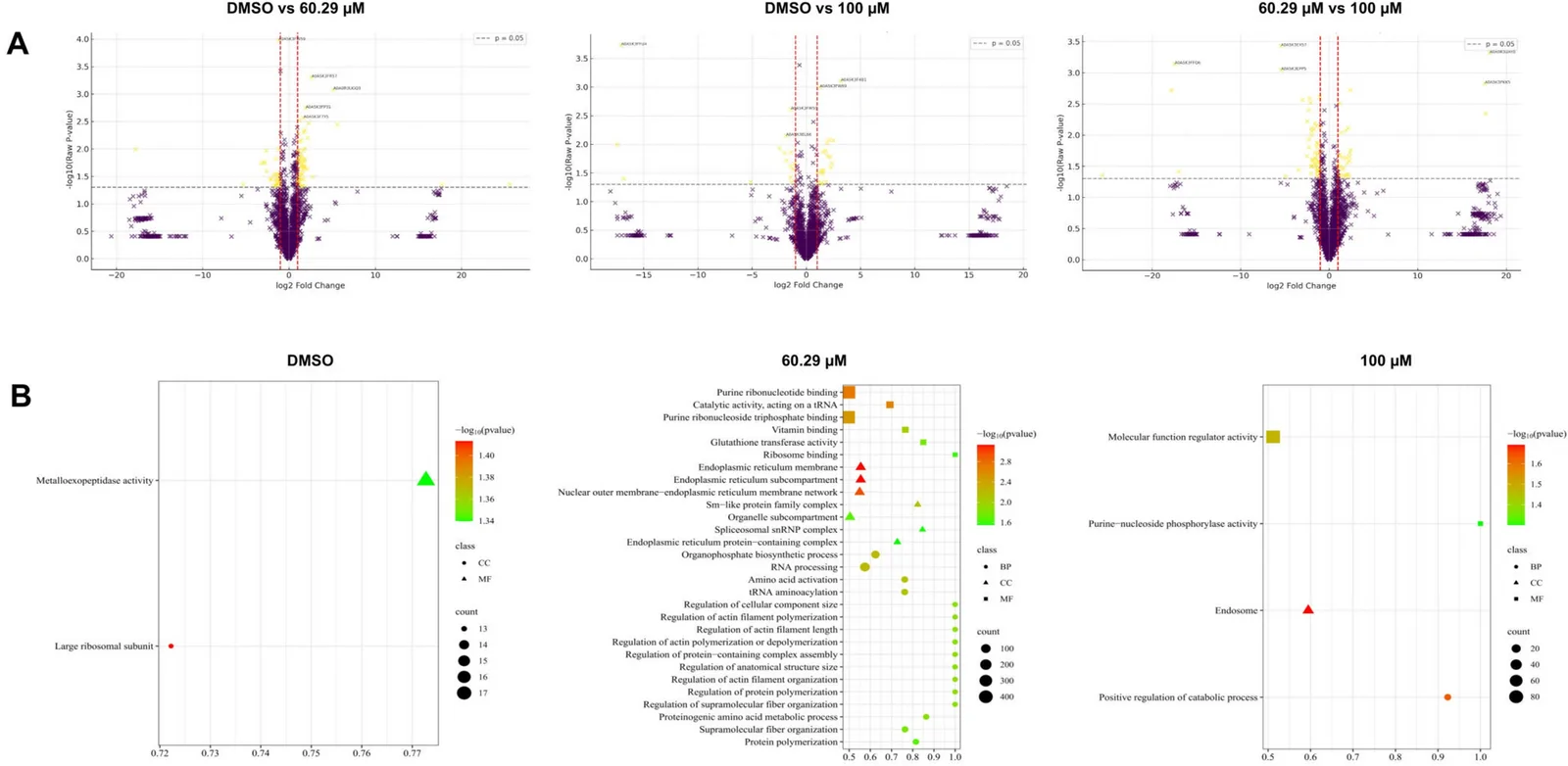
Differential protein abundance and functional enrichment analysis across experimental conditions. Overview of differentially abundant proteins among the DMSO control, 60.29 µM, and 100 µM treatment conditions, together with Gene Ontology (GO) functional enrichment analyses. **A** Volcano plots showing pairwise comparisons between DMSO vs. 60.29 µM, DMSO vs. 100 µM, and 60.29 µM vs. 100 µM conditions. Proteins exhibiting statistically significant differential abundance (p < 0.05) with a fold change (FC) > 1 are highlighted in yellow. **B** Bubble plot summarizing GO term enrichment for each experimental condition. Each bubble represents a significantly enriched GO term, grouped on the y-axis by category—Biological Process (BP), Molecular Function (MF), and Cellular Component (CC). The x-axis indicates the enrichment score. Bubble color denotes statistical significance (− log10 p-value), according to the scale shown, while bubble size corresponds to the number of proteins annotated to each term. The bubble shape distinguishes the GO categories

In summary, although simvastatin at concentrations of 60.29 µM and 100 µM did not elicit a clear proteomic signature of cell death in *M. corti*, it triggered a dose-dependent response involving proteins associated with homeostasis, metabolism, and stress-related processes. These findings are consistent with the considerable physiological adaptability described for cestodes in response to environmental and host-derived challenges [17]. Importantly, the therapeutic relevance of a molecular target does not necessarily depend on the induction of cell death but may also derive from disrupting pathways required for parasite fitness and survival. In this regard, the modulation of compensatory pathways observed following simvastatin exposure supports the biological relevance of the mevalonate pathway in *M. corti*. Furthermore, pharmacological inhibition of this pathway with simvastatin (50–150 µM) has previously been shown to reduce motility and viability of TT larvae *in vitro* [12], indicating that mevalonate pathway inhibition imposes a measurable biological cost on the parasite. Together, these findings support HMG-CoA reductase as a potential therapeutic target for larval cestodiases. Future studies should investigate strategies to enhance pathway inhibition, including optimized statin analogs, combination approaches, and *in vivo* assessments to determine the translational impact of mevalonate pathway inhibition on larval cestodiases.

## Supplementary Information

Additional file 1.

Additional file 2.

Additional file 3.

Additional file 4.

Additional file 5.

Additional file 6.

Additional file 7.

## Data Availability

The mass spectrometry proteomics data have been deposited to the MassIVE repository (UCSD) under the dataset identifier MSV000101236.
